# Microwave assisted synthesis of triazoloquinazolinones and benzimidazoquinazolinones

**DOI:** 10.1186/1860-5397-3-11

**Published:** 2007-03-05

**Authors:** Aboul-Fetouh E Mourad, Ashraf A Aly, Hassan H Farag, Eman A Beshr

**Affiliations:** 1 Chemistry Department, Faculty of Science, El-Minia University, 61519-El-Minia, Egypt; 2 Medicinal Chemistry Department, Faculty of Pharmacy, Assiut University, Assiut, Egypt; 3 Medicinal Chemistry Department, Faculty of Pharmacy, El-Minia University, 61519-El-Minia, Egypt

## Abstract

**Background:**

Benzimidazoquinazolinones and related quinazolines are classes of heterocycles that are of considerable interest because of the diverse range of their biological properties. Although numerous classes of quinazolines have been conventionally synthesized, their syntheses have been suffered due to the multiple steps that sometimes have described in their preparation and also their chemical transformations that have been taken hours or even days to be completed. However, microwave energy can offer numerous benefits for performing synthesis of organic compounds including reduced pollution, increased reaction rates, yield enhancements, and cleaner chemistries.

**Results:**

Synthesis of a series of triazoloquinazolinones and benzimidazoquinazolinones has been achieved under microwave irradiation. The products were obtained in nearly quantitative yields within few minutes during the reaction of aromatic aldehydes with 5-amino-1(*H*)-1,2,4-triazole (or 2-aminobenzimidazole) and dimedone in DMF.

**Conclusion:**

Microwave irradiation can be used as a facile and general method for the construction of a wide variety of triazoloquinazolinones and benzimidazoquinazolinones. The reaction involves a three component condensation (with potential for combinatorial work) being carried out with almost productive yields by microwave irradiation and considerably shortened reaction time.

## Background

Condensed heterocyclic systems with a partially or a completely reduced pyrimidine nucleus are of interest, since they display valuable pharmaceutical activities. [1–2] There has been an increasing interest in the chemistry of 4(3*H*)-quinazolinones because of their biological significance. Many of them show antifungal, [3] antibacterial, [4] anticancer, [5] anti-inflammatory, [6] anticonvulsant, [7] and antiproliferative activities as well as inhibitory effects for thymidylate synthase and poly-(*ADP*-ribose) polymerase (*PARP*). [8] An interesting method for quinazoline synthesis involved [5+1] annulation during the reaction of β-dicarbonyl compounds with 2-ethoxymethyleneaminonitriles. [9] A general route to prepare 5,10-dihydro-[1,2,4]-triazolo-[5,1-*b*]-quinazolines included the reaction of benzophenone and 1-ureidoethylidene-hydrazones with a mixture of triphenylphosphine, carbon tetrachloride, and triethylamine in dichloromethane. [10] Other triazoloquinazolines were obtained from 2'-azidoacetophenone and as well as 2-azidobenzonitrile. [11] Quinazolines were also obtained in moderate yields by an intermolecular reductive *N*-heterocyclization of 2-nitro-benzaldehydes or 2-nitrophenyl ketones with formamide catalyzed by a combination of PdCl_2_(PPh_3_)_2_ in the presence of MoCl_5_. [12] Palladium-catalyzed cyclocarbonylation of *o*-iodoanilines with heterocumulenes is a further route to 4(3*H*)-quinazolinone derivatives albeit one that requires elevated reaction pressures. [13] Benzimidazoquinazolinones and related quinazolines are classes of heterocycles that are of considerable interest because of the diverse range of their biological properties, including anticancer, [14] anti-inflammatory, [15] anticonvulsant, [16] and antidituric, [17] activities. Moreover, the imidazoquinazolinones have the activity to afford agents capable of lowering the blood pressure of experimental animals. [18] Additionally, naturally occurring substances as known such as *clonidine* and *moxonidine* or belong to this class exhibit antihypertensive activity mediated by α-adrenergic and/or imidazoline receptors. [19] Recently, it was reported on an efficient strategy for the preparation of structural variant of imidazoquinazolinones with three-point diversity. [20]

The use of microwaves in organic synthesis has increased dramatically in the last years, receiving widespread acceptance and becoming an indispensable tool. [21] Microwave technology has become a powerful tool in organic synthesis, since by employing this technique it is generally possible to prepare organic compounds very fast, with high purity and better yields compared to other more conventional methods. [22–24] Additionally, in the search for economic and environmentally friendly synthetic methods, one-pot syntheses could offer a significant step ahead. Since, the aim of our research has concerned on achieving reasonable yields of the synthesized heterocyclic compounds which might have prospective biological and pharmaceutical activities, we have synthesized many heterophanes from cycloadditions of alkenyl [2.2]paracyclophanes. [25] Previously, we also prepared various heterocycles such as triazoloes, [26] acridinones, [27] and pyrazolidines [28]. In this publication our goal is to synthesize known and/or new of fused quinalzolines, namely triazoloquinazolinones and benzimidazoquinazolinones using microwave irradiation.

## Results and Discussion

**Scheme 1:**
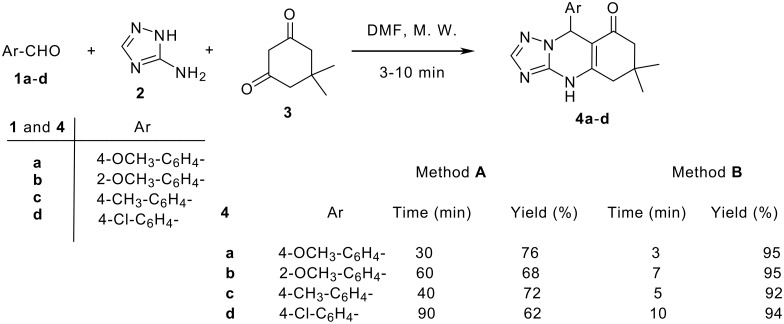
Synthesis of triazoloquinazolinones **4a-d** *via* microwave irradiation.

The synthesis of 9-aryl-dimethyl-5,6,7,9-tetrahydro-1,2,4-triazolo-[5,1-*b*]quinazolin-8(4*H*)-ones by refluxing equimolar amounts of aromatic aldehydes (**1**, C_6_H_5_-, 4-OCH_3_-C_6_H_4_-, 4-NMe_2_-C_6_H_4_-, 4-Cl-C_6_H_4_- and 4-NO_2_C_6_H_4_-), 5-amino-1(*H*)-1,2,4-triazole (**2**) and dimedone (**3**) in DMF or methanol has been reported previously. [29–30] Fortunately, the microwave irradiation for the reaction containing the starting materials **1a**-**d**, **2** and **3**, afforded within a few minutes compounds **4a**-**d** in nearly quantitative yields (Scheme 1). The products **4a**,**d** are the same as obtained by the reported conventional procedure [29–30]. We chose another two aromatic aldehydes **1b**,**c** and accordingly, we obtained the corresponding triazoloquinazolinones **4b**,**c** as shown in Scheme 1. The structure of compounds **4b**,**c** is in accord with the spectral data in IR, ^1^H NMR, ^13^C NMR and mass spectral data in addition to elemental analyses (see Supporting Information File 1 for full experimental data). The IR spectra of **4b**,**c** revealed an intense carbonyl absorption band at ν_max_ 1655-1650 cm^-1^ and a broad band was found in the range from 3390 to 3400 cm^-1^ due to the presence of a NH group. Analysis of the ^1^H NMR spectra of **4a**-**d** provided answers on the chemoselectivity of the cyclocondensation. The δ values of the most carbon signals could be determined and assigned to the corresponding carbon atoms. For example, the ^13^C NMR spectrum of compound **4b** revealed fourteen distinctive carbon signals at *δ_C_* 192.0, 156.6, 150.1, 148.90, 133.8, 130.0, 128.8, 128.0, 127.6, 126.8, 113.0, 57.2, 98.8 and 49.8 corresponding to *C*-8, Ar-*C*-4a, *C*H-3, Ar-*C*-2^'^, Ar-C-1^'^, Ar-*C*H-6^'^, Ar-*C*H-3^'^, Ar-*C*H-4^'^, *C*-5a, Ar-*C*H-5^'^, C-8a, O*C*H_3_, *C*H-9, and *C*-7 respectively. The remaining carbon signals in compound **4b** resonated at δ = 28.4 and 26.8 ppm could be assigned to the two methyl carbons (see Supporting Information File 1 for full experimental data).

**Scheme 2:**
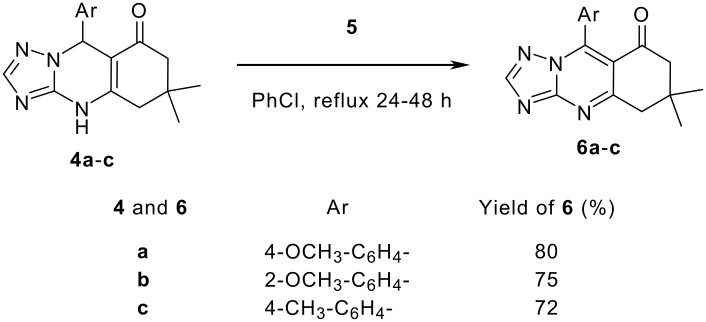
Oxidation of tetrahydrotriazoloquinazolinones.

To test the reactivity of compounds **4a**-**c** towards oxidation, we refluxed a mixture of 2,3,5,6-tetrachloro-1,4-benzoquinone (**5**) and **4a**-**c** in cholorobenzene (Scheme 2). The reaction proceeded after long time of refluxing to give compounds **6a**-**c** in good yields. The structure feature of the products **6a**-**c** was confirmed by the IR, mass, ^1^H NMR and ^13^C NMR spectra in addition to elemental analyses. The IR spectra did not show any absorption due to the presence of NH group and this was also noted in the ^1^H NMR spectra. The disappearance of 9-H in the ^1^H NMR spectra and the appearance of C-5a in the ^13^C NMR spectra as an azomethine carbon signal at δ = 154.8–155.2 indicated that only the NH and 9-H protons were removed from the starting materials **4a**-**c** (Scheme 2). The structure of the compounds in hand was confirmed to be that of dihydro-5*H*-[1,2,4]triazolo [5,1-*b*]-quinazolin-8-ones (Scheme 2).

Microwave irradiation can be used to induce the synthesis of another class of fused quinazolinones, namely, benzimidazoquinazolinones **8a**-**e**. Indeed, we succeeded to obtain compounds **8a**-**e** from the reaction of **7** with the aromatic aldehydes **1a**-**e** and dimedone (**3**) in DMF. We used the normal method of refluxing (method A) and compared it with the microwave irradiation (method B).

**Scheme 3:**
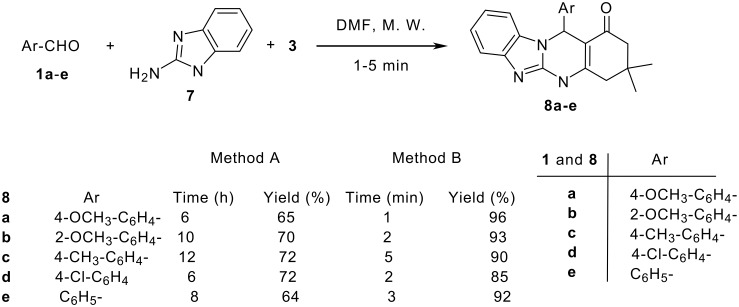
Synthesis of benzimidazoquinazolinones **8a-e** *via* microwave irradiation.

The results in Scheme 3 indicated the isolation of products **8a**-**e** by the two methods, however, in different yields. It is worthwhile to note that the reaction time was reduced in the case of microwave irradiation method (Scheme 3). The mass, IR and NMR spectra and elemental analyses unambiguously confirmed the structure of **8a**-**e**. For example, the mass spectroscopy and elemental analysis proved the molecular formula of compound **8a** as C_23_H_23_N_3_O_2_. The IR spectrum indicated the presence of the NH and the C=O groups at 3380 and 1660 cm^-1^, respectively. The ^1^H NMR spectrum showed a singlet at δ = 11.40 for NH, two double-doublets at δ = 7.60 and 7.40 (*J* = 8.2, 1.2 Hz) for four protons (each for two protons). The aliphatic proton (5-H) resonated as a singlet in the ^1^H NMR spectrum of **8a** at δ = 6.08, whereas the methoxy protons absorb as a singlet at δ = 3.95. Two singlets appeared at δ = 1.06 and 0.94 ppm, assigned to the two methyl protons, and the resonance for one methylene group (CH_2_-9) was observed as a singlet at δ = 2.46, whereas the other methylene (CH_2_-7) appeared as two doublets at δ = 2.20 (*J* = 15.8 Hz) and 2.05 (*J* = 15.8 Hz) (see Supporting Information File 1 for full experimental data). The ^13^C NMR spectrum supported the ^1^H NMR spectral data of the related compound. The spectral data obtained and the elemental analyses of compounds **8a**-**e** proved the structure of benzimidazoquinazolinones (see Scheme 3 and Supporting Information File 1 for full experimental data).

In conclusion, microwave irradiation can be used as a facile and general method for the construction of a variety of triazoloquinazolinones and benzimidazoquinazolinones. The reaction involves a three component condensation (with potential for combinatorial work) being carried out with almost productive yields by microwave irradiation and considerably shortened reaction time (This conclusion was suggested by the referee.).

## Supporting Information

File 1 Experimental detail data which includes experimental detail of the spectral instruments, elemental analyzer along with microwave device. This file describes the type of preparative thin layer chromatography used for purification. Additionally, this file contains a detail of methods A and B of triazoloquinazolinones and benzimidazoquinazolinones synthesis along with the oxidation procedure.
