# Supplementary material for: Microwave assisted synthesis of triazoloquinazolinones and benzimidazoquinazolinones
Source: Beilstein J Org Chem. 2007 Mar 5;3:11. doi: 10.1186/1860-5397-3-11 (PMC1847824; doi:10.1186/1860-5397-3-11)
Supplement: File 1 — Experimental detail data which includes experimental detail of the spectral instruments, elemental analyzer along with microwave device. This file describes the type of preparative thin layer chromatography used for purification. Additionally, this file contains a detail of methods A and B of triazoloquinazolinones and benzimidazoquinazolinones synthesis along with the oxidation procedure. [file Beilstein_J_Org_Chem-03-11-s001.doc]

**Additional file #1**

**3. Experimental**

***3.1. General***

Melting points are uncorrected. IR spectra were obtained on Shimadzu 470 spectrophotometer using potassium bromide pellets. ^1^H NMR (400.134 MHz) and ^13^C NMR (100.6 MHz) spectra were measured on Bruker AM 400 with TMS as an internal standard. Coupling constants are expressed in Hz. Mass spectra were recorded on a Finnigan MAT 8430 instrument at 70 eV. Elemental analyses were carried out in the Microanalysis Center of the Institut für Anorganische Chemie, Technische Universität Braunschweig. For preparative thin layer chromatography (PLC), glass plates (20 x 48 cm) were covered with slurry of silica gel Merck PF_254_ and air-dried using the solvents listed for development. Zones were detected by quenching of indicator fluorescence upon exposure to 254 nm light; elution of the different bands with toluene afforded the pure products. The microwave device (household oven) was operated at 80-100 ^o^C.

**Method A**

A mixture of dimedone (**2**, 1 mmol, 140 mg), 5-amino-1*H*-1,2,4-triazole (**3**, 1 mmol, 84 mg) and the corresponding aldehyde **1b**,**c** (1 mmol) in DMF (20 ml) was heated under reflux for nearly 1 h (the reaction was followed by TLC analysis). The solvent was evaporated under vacuum to half of its volume and the product obtained was recrystallized from the stated solvents.

**Method B**

On applying the same procedure mentioned before, the equivalent amounts of **1a**-**d**, **2** and **3** were good mixed in DMF (5 ml). The mixture was irradiated in microwave for 3-10 min (80-100 ^o^C). On cooling at room temperature, the precipitates of the products **4a**-**d** were filtered and recrystallized from the stated solvents.

**Method A**

***Synthesis of 9-(aryl)-6,6-dimethyl-5,6,7,9-tetrahydro-4H-[1,2,4]-triazolo-[5,1-b]-quinazolin-8-ones (4b****,****c)***

***9-(2\-Methoxyphenyl)-6,6-dimethyl-5,6,7,9-tetrahydro-4H-[1,2,4]-triazolo-[5,1-b]-quinazolin-8-one (4b):*** Compound **4b** (220 mg, 68%) was obtained as colorless crystals (DMF/ethanol). M.p. 240-2 ˚C. Anal. Calcd for C_18_H_20_N_4_O_2_ (324.39): C, 66.65; H, 6.21; N, 17.27. Found: C, 66.58; H, 6.16; N, 17.20. IR (KBr): 3400 (m, NH), 3035-2990 (m, Ar-CH), 2980-2960 (m, Aliph-CH), 1655 (s, CO), 1600 (s, C=N), 1580 (s, C=C) cm^-1^. ^1^H NMR (400 MHz, DMSO-*d_6_*): δ = 11.05 (s , 1 H, NH), 7.65 (s, 1 H, 3-H), 7.20-6.80 (m, 4 H, Ar-H), 6.10 (s, 1 H, 9-H), 3.90 (s, 3 H, OCH_3_), 2.40 (s, 2 H, 7-H), 2.25 (d, *J* =16.1 Hz, 1 H, 5-H), 2.10 (d, *J* = 16.1 Hz, 1 H, 5\-H), 1.01 (s, 3 H, CH_3_), 0.99 (s, 3 H, CH_3_). ^13^C NMR (400 MHz, DMSO-*d_6_*): δ = 192.0 (C-8), 156.6 (Ar-C-4a), 150.1 (CH-3), 148.9 (Ar-C-2\), 133.8 (Ar-C-1**\**), 130.0 (Ar-CH-6\), 128.8 (Ar-CH-3\), 128.0 (Ar-CH-4**?**), 127.6 (C-5a), 126.8 (Ar-CH-5**\**), 113.0 (C-8a), 98.8 (CH-9), 57.2 (OCH_3_), 49.8 (C-7), 40.1 (C-5), 32.1 (C-6), 28.4 (CH_3_), 26.8 (CH_3_). EI-MS: *m*/*z* (%) = 324 (100) [M^+^], 309 (22), 294 (24), 263 (18), 258 (20), 186 (30), 170 (14), 128 (24), 108 (34), 77 (24).

***9-(4\-Methylphenyl)-6,6-dimethyl-5,6,7,9-tetrahydro-4H-[1,2,4]-triazolo-[5,1-b]-quinazolin-8-one (4c)*:** Compound **4c** (222 mg, 72%) was obtained as colorless crystals (acetone). M.p. 280-2 ºC. Anal. Calcd for C_18_H_20_N_4_O (308.39): C, 70.11; H, 6.54; N, 18.17. Found: C, 69.93; H, 6.52; N, 18.08. IR (KBr): 3390 (m, NH), 3030-2980 (m, Ar-CH), 2970-2856 (m, Aliph-CH), 1650 (s, CO), 1608 (s, C=N), 1590 (m, C=C) cm^-1^. ^1^H NMR (400 MHz, DMSO-*d_6_*): δ = 11.00 (s , 1 H, NH), 7.70 (s, 1 H, 3-H), 7.10 (dd, *J* = 8.2, 1.2 Hz, 2 H, Ar-H), 6.84 (dd, *J* = 8.1, 1.2 Hz, 2 H, Ar-H), 5.98 (s, 1 H, 9-H), 2.38 (s, 3 H, CH_3_), 2.36 (s, 2 H, 7-H), 2.22 (d, *J* = 16.2 Hz, 1 H, 5-H), 2.08 (d, *J* = 16.1 Hz, 1 H, 5\-H), 1.00 (s, 3 H, CH_3_), 0.98 (s, 3 H, CH_3_). ^13^C NMR (400 MHz, DMSO-*d_6_*): δ = 192.4 (C-8), 157.0 (Ar-C-4a), 150.8 (CH-3), 132.8 (Ar-C-4\), 130.0 (Ar-C-1**\**), 128.2 (2 Ar-CH, CH-2\, -6\), 128.0 (2 Ar-CH, CH-3\, -5\), 127.2 (C-5a), 113.4 (C-8a), 98.4 (CH-9), 49.4 (C-7), 40.2 (C-5), 32.6 (Ph-CH_3_), 32.0 (C-6), 28.4 (CH_3_), 26.4 (CH_3_). EI-MS: *m*/*z* (%) = 308 (100) [M^+^], 293 (12), 278 (20), 262 (14), 186 (40), 144 (20), 92 (32), 77 (22).

**Method B**

Compounds **4a**-**d** were obtained by the yields indicated in Scheme 1. The spectroscopic data of compounds **4a**,**d** are in a good agreement with those reported in reference, [20] whereas for compounds **4b**,**c** the same values were found as mentioned above.

***3.2. Oxidation of 4a-c by 5: Synthesis of 9-(aryl)-6,6-dimethyl-6,7-dihydro-5H-[1,2,4]-triazolo-[5,1-b]quinazolin-8-ones (6a****-****c)***

**General procedure**

A mixture of compounds **4a-c** (1 mmol) and **5** (1.2 mmol, 296 mg) in chlorobenzene (30 ml) was stirred at reflux temperature for 18-24 h (the reaction was followed by TLC analysis). The precipitate of dihydro-**5** was filtered and washed several times with chloroform (100 ml). The solvents were evaporated under vacuum and the residue was purified by preparative plates chromatography (silica gel) using toluene/ethyl acetate 2:1 to give the oxidized products **6a**-**c**, which were recrystallized from the stated solvents.

***9-(4\-Methoxyphenyl)-6,6-dimethyl-6,7-dihydro-5H-[1,2,4]triazolo[5,1-b]-quinazolin-8-one (6a):*** Compound **6a** (258 mg, 80 %) was obtained as yellow crystals (methanol).

M.p. 290-2˚C. IR (KBr): 3028-2986 (m, Ar-CH), 2960-2860 (m, Aliph-CH), 1660 (s, CO), 1620 (s, C=N), 1594 (m, C=C) cm^-1^. ^1^H NMR (400 MHz, DMSO-*d_6_*): δ = 7.64 (s, 1 H, 2-H), 7.45 (dd, *J* = 8.7, 1.2 Hz, 2 H, Ar-H), 7.10 (dd, *J* = 8.6, 1.2 Hz, 2 H, Ar-H), 3.95 (s, 3 H, OCH_3_), 2.48 (s, 2 H, 7-H), 2.25 (d, *J* = 16.1 Hz, 1 H, H-5), 2.08 (d, *J* = 16.1 Hz, 1 H, H-5\), 1.00 (s, 3 H, CH_3_), 0.96 (s, 3 H, CH_3_). ^13^C NMR (400 MHz, DMSO-*d_6_*): δ = 192.0 (C-8), 160.0 (Ar-C-4\), 154.8 (C-5a), 150.4 (CH-3), 147.0 (C-4a), 142.0 (C-9), 130.8 (Ar-C-1**\**), 128.0 (2 Ar-CH, CH-2\, -6\), 127.6 (2 Ar-CH, CH-3\, -5\), 110.0 (C-8a), 57.6 (OCH_3_), 50.0 (C-7), 40.0 (C-5), 32.0 (C-6), 28.4 (CH_3_), 26.6 (CH_3_). EI-MS: *m*/*z* (%) = 322 (100) [M^+^], 307 (18), 292 (16), 262 (20), 184 (22), 142 (18), 108 (60), 77 (34). Anal. Calcd for C_18_H_18_N_4_O_2_ (322.37) C, 67.07; H, 5.63; N, 17.38. Found: C, 67.25; H, 5.62; N, 17.30.

***9-(2\-Methoxyphenyl)-6,6-dimethyl-6,7-dihydro-5H-[1,2,4]triazolo-[5,1-b]-quinazolin-8-one (6b):*** Compound **6b** was obtained (242 mg, 75%) as yellow crystals (methanol).

M.p. 276-8˚C. IR (KBr): 3034-2990 (m, Ar-CH), 2970-2862 (m, Aliph-CH), 1658 (s, CO), 1616 (s, C=N), 1590 (m, C=C) cm^-1^. ^1^H NMR (400 MHz, DMSO-*d_6_*): δ = 7.60 (s, 1 H, 2-H), 7.40-7.28 (m, 4 H, Ar-H), 3.92 (s, 3 H, OCH_3_), 2.45 (s, 2 H, 7-H), 2.25 (d, *J* =16.1 Hz, 1 H, 5-H), 2.10 (d, 1 H, *J* = 16.1 Hz, 5\-H), 1.01 (s, 3 H, CH_3_), 0.99 (s, 3 H, CH_3_). ^13^C NMR (400 MHz, DMSO-*d_6_*): δ = 191.8 (C-8), 160.0 (Ar-C-2\), 155.0 (C-5a), 150.0 (CH-3), 146.8 (C-4a), 141.8 (C-9), 132.8 (Ar-C-1**\**), 128.0, 127.8, 127.2, 126.8 (4 Ar-CH, CH-3\, -4\, 5\, -6\), 109.8 (C-8a), 58.6 (OCH_3_), 49.6 (C-7), 40.2 (C-5), 32.2 (C-6), 28.2 (CH_3_), 26.4 (CH_3_). EI-MS: *m*/*z* (%) = 322 (100) [M^+^], 306 (24), 292 (20), 260 (18), 184 (18), 142 (24), 108 (64), 77 (38). Anal. Calcd for C_18_H_18_N_4_O_2_ (322.37): C, 67.07; H, 5.63; N, 17.38. Found: C, 67.15; H, 5.60; N, 17.35.

***9-(4\-Methylphenyl)-6,6-dimethyl-6,7-dihydro-5H-[1,2,4]triazolo-[5,1-b]-quinazolin-8-one (6c):*** Compound **6c** (220 mg, 72%) was obtained as yellow crystals (methanol). M.p. 310-12˚C. IR (KBr): 3020-2990 (m, Ar-CH), 2960-2870 (w, Aliph-CH), 1656 (s, CO), 1610 (s, C=N), 1594 (m, C=C) cm^-1^. ^1^H NMR (400 MHz, DMSO-*d_6_*): δ = 7.65 (s, 1 H, 2-H), 7.10 (dd, *J* = 8.2, 1.2 Hz, 2 H, Ar-H), 6.65 (dd, *J* = 8.2, 1.2 Hz, 2 H, Ar-H), 2.40 (s, 2 H, 7-H), 2.34 (s, 3 H, CH_3_), 2.22 (d, *J* = 15.8 Hz, 1 H, 5-H), 2.12 (d, *J* = 16.0 Hz, 1 H, 5\-H), 0.99 (s, 6 H, 2 CH_3_). ^13^C NMR (400 MHz, DMSO-*d_6_*): δ = 191.8 (C-8), 155.2 (C-5a), 150.6 (CH-3), 146.6 (C-4a), 141.6 (C-9), 132.6 (Ar-C-4\), 132.0 (Ar-C-1**\**), 128.2 (2 Ar-CH, CH-2\, -6\), 128.0 (2 Ar-CH, CH-3\, -5\), 110.0 (C-8a), 49.0 (C-7), 40.6 (C-5), 32.8 (Ar-CH_3_), 32.0 (C-6), 28.2 (CH_3_), 26.0 (CH_3_). EI-MS: *m*/*z* (%) = 306 (100) [M^+^], 292 (45), 276 (22), 262 (10), 184 (54), 142 (16), 92 (36), 77 (24). Anal. Calcd for C_18_H_18_N_4_O (306.36): C, 70.57; H, 5.92; N, 18.29. Found: C, 70.70; H, 5.90; N, 18.25.

***3.3. Synthesis of 5-(aryl)-8,8-dimethyl-5,8,9,10-tetrahydro-7H-4b,10-,11-triaza-benzo-[b]-fluoren-6-ones (8a-e): General procedure: Method A:*** A mixture of dimedone (**2**, 1 mmol, 140 mg), 2-aminobenzimidazole (**7**, 1 mmol, 133 mg) and the appropriate aldehyde (**1a**-**e**, 1 mmol) in absolute DMF (20 ml) was heated under reflux for 6-12 h. During heating, the products were precipitated. After cooling at room temperature the precipitates were filtered, washed with hot ethanol and recrystallized from the stated solvents.

**Method B**

The same above mentioned mixture **1a**-**e**, **2** and **7** in DMF (5 ml) was irradiated in the microwave oven for 1-5 min. The formed precipitates, after cooling at room temperature, were filtered, washed with hot ethanol and recrystallized from the stated solvents.

**Method A**

***5-(4\-Methoxyphenyl)-8,8-dimethyl-5,8,9,10-tetrahydro-7H-4b,10,11-triaza-benzo[b]-fluoren-6-one (8a):*** Compound **8a** was obtained as colorless crystals (242 mg, 65%). M.p. 318-20ºC (DMF/ethanol). IR (KBr): 3380 (m, NH), 3040-2990 (m, Ar-CH), 2870-2830 (m, Aliph-CH), 1660 (s, CO), 1610 (C=N), 1580 (m, C=C) cm^-1^. H NMR (400 MHz, DMSO-*d_6_*): δ = 11.40 (s, 1 H, NH), 7.60 (dd, *J* = 8.2, 1.2 Hz, 2 H, Ar-H), 7.40 (dd, *J* = 8.0, 1.2 Hz, 2 H, Ar-H), 7.10-6.80 (m, 4 H, Ar-H), 6.08 (s, 1 H, 5-H), 3.95 (s, 3 H, OCH_3_), 2.46 (s, 2 H, CH_2_), 2.20 (d, *J* = 15.8 Hz, 1 H, 7-H), 2.05 (d, *J* = 15.8 Hz, 1 H, 7\-H), 1.06 (s, 3 H, CH_3_), 0.94 (s, 3 H, CH_3_). ^13^C NMR (400 MHz, DMSO-*d_6_*): δ = 190.0 (C-6), 158.0 (Ar-C-4\), 141.0 (C-10a), 140.0 (C-9a), 134.8, 134.4 (bz-C), 131.0 (Ar-C-1\), 128.8, 128.0, 127.8, 127.0, 126.2, 126.0, 124.8, 124.4 (Ar-CH), 110.0 (C-6a), 98.4 (CH-5), 58.4 (OCH_3_), 48.6 (C-7), 40.1 (C-9), 32.0 (C-8), 28.4 (CH_3_), 26.8 (CH_3_). EI-MS: *m*/*z* (%) = 374 (40) [M+1], 373 (100) [M^+^], 358 (30), 342 (24), 328 (18), 265 (60), 182 (20), 108 (34), 77 (20). Anal. Calcd for C_23_H_23_N_3_O_2_ (373.46): C, 73.97; H, 6.21; N, 11.25. Found: C, 73.80; H, 6.18; N, 11.20.

***5-(2\-Methoxyphenyl)-8,8-dimethyl-5,8,9,10-tetrahydro-7H-4b,10,11-triaza-benzo[b]-fluoren-6-one (8b):*** Compound **8b** (261 mg, 70%) was obtained as colorless crystals (DMF/ethanol). M.p. 325-7 ºC. IR (KBr): 3400 (m, NH), 3040-2985 (m, Ar-CH), 2970-2860 (m, Aliph-CH), 1621 (s, CO), 1608 (s, C=N), 1590 (s, C=C) cm^-1^. ^1^H NMR (400 MHz, DMSO-*d_6_*): δ = 11.60 (s, 1 H, NH), 7.40-7.20 (m, 4 H, Ar-H), 7.10-6.80 (m, 4 H, Ar-H), 6.00 (s, 1 H, 5-H), 3.92 (s, 3 H, OCH_3_), 2.44 (s, 2 H, CH_2_), 2.14 (d, *J* = 16.0 Hz, 1 H, 7-H), 2.07 (d, *J* = 16.0 Hz, 1 H, 7\-H), 1.06 (s, 3 H, CH_3_), 0.96 (s, 3 H, CH_3_). ^13^C NMR (400 MHz, DMSO-*d_6_*): δ = 190.2 (C-6), 156.8 (Ar-C-2\), 141.2 (C-10a), 140.4 (C-9a), 134.8, 134.4 (bz-C), 132.0 (Ar-C-1\), 128.6, 127.8, 127.4, 127.0, 126.0, 125.8, 124.6, 124.4 (Ar-CH), 110.6 (C-6a), 98.2 (CH-5), 56.9 (OCH_3_), 48.4 (C-7), 40.0 (C-9), 32.4 (C-8), 28.2 (CH_3_), 26.6 (CH_3_). EI-MS: *m*/*z* (%) = 373 (100) [M^+^], 356 (28), 340 (20), 310 (20), 264 (58), 234 (32), 182 (18), 108 (30), 77 (24). Anal. Calcd for C_23_H_23_N_3_O_2_ (373.46): C, 73.97; H, 6.21; N, 11.25. Found: C, 73.79; H, 6.18; N, 11.23.

***5-(4\-Methylphenyl)-8,8-dimethyl-5,8,9,10-tetrahydro-7H-4b,10,11-triaza-benzo[b]-fluoren-6-one (8c):*** Compound **8c** (257 mg, 72%) was obtained as colorless crystals (acetone). M.p. 330-2 ^o^C. IR (KBr): 3380 (m, NH), 3028-2986 (m, Ar-CH), 2976-2860 (m, Aliph-CH), 1660 (s, CO), 1616 (s, C=N), 1594 (m, C=C) cm^-1^. ^1^H NMR (400 MHz, DMSO-*d_6_*): δ = 11.40 (s, 1 H, NH), 7.30-7.00 (m, 5 H, Ar-H), 6.90-6.70 (m, 3 H, Ar-H), 6.00 (s, 1 H, 5-H), 2.40 (s, 2 H, CH_2_), 2.38 (s, 3 H, CH_3_), 2.16 (d, *J* = 15.8 Hz, 1 H, 7-H), 2.07 (d, *J* = 15.8 Hz, 1 H, 7\-H), 1.08 (s, 3 H, CH_3_), 0.99 (s, 3 H, CH_3_). ^13^C NMR (400 MHz, DMSO-*d_6_*): δ = 190.4 (C-6), 141.4 (C-10a), 140.6 (C-9a), 136.0 (Ph-C-CH_3_) 134.6, 134.2 (bz-C), 130.0 (Ar-C-1\), 128.8, 128.4, 128.0, 127.6, 127.2, 126.8, 126.6, 126.2 (Ar-CH), 110.4 (C-6a), 98.6 (CH-5), 48.0 (C-7), 40.2 (C-9), 32.8 (Ph-CH_3_), 32.2 (C-8), 28.4 (CH_3_), 26.8 (CH_3_).

EI-MS: *m*/*z* (%) = 357 (100) [M^+^], 342 (28), 264 (70), 248 (14), 264 (22), 236 (14), 220 (16), 182 (18), 108 (34), 77 (20). Anal. Calcd for C_23_H_23_N_3_O (357.46): C, 77.28; H, 6.49; N, 11.76. Found: C, 77.16; H, 6.46; N, 11.74.

***5-(4\-Chlorophenyl)-8,8-dimethyl-5,8,9,10-tetrahydro-7H-4b,10-,11-triaza-benzo[b]-fluoren-6-one (8d):*** Compound **8d** (271 mg, 72%) was obtained as colorless crystals (ethyl acetate). M.p. 340 ^o^C. IR (KBr): 3400 (m, NH), 3020-2990 (m, Ar-CH), 2980-2850 (m, Aliph-CH), 1654 (s, CO), 1610 (s, C=N), 1594 (s, C=C) cm^-1^. ^1^H NMR (400 MHz, DMSO-*d_6_*): δ = 11.30 (s, 1 H, NH), 7.10-6.40 (m, 8 H, Ar-H), 5.84 (s, 1 H, 5-H), 2.34 (s, 2 H, CH_2_), 2.18 (d, *J* = 16.0 Hz, 1 H, 7-H), 2.05 (d, *J* = 16.0 Hz, 1 H, 7\-H), 1.00 (s, 6 H, 2 CH_3_). ^13^C NMR (400 MHz, DMSO-*d_6_*): δ = 190.0 (C-6), 141.0 (C-10a), 140.4 (C-9a), 134.0, 133.8 (Ar-C), 128.8 (Ar-C-1\), 127.8, 127.4, 126.8, 126.4, 126.0, 124.4, 122.8, 122.0 (Ar-CH), 110.0 (C-6a), 98.8 (CH-5), 48.2 (C-7), 40.2 (C-9), 32.4 (C-8), 28.6 (CH_3_), 26.6 (CH_3_). EI-MS: *m*/*z* (%) = 379(38) [M+2], 378 (60) [M+1], 377 (100) [M^+^], 362 (18), 346 (16), 312 (20), 310 (18), 264 (30), 236 (22), 234 (26), 192 (24), 77 (16). Anal. Calcd for C_22_H_20_ClN_3_O (377.88): C, 69.93; H, 5.33; N, 11.12. Found: C, 69.86; H, 5.30; N, 11.10.

***5-Phenyl-8,8-dimethyl-5,8,9,10-tetrahydro-7H-4b,10,11-triaza-benzo[b]-fluoren-6-one (8e):*** Compound **8e** was obtained (222 mg, 65%) as colorless crystals (acetone). M.p. 322-4ºC. IR (KBr): 3380 (m, NH), 3020-2990 (m, Ar-CH), 2970-2870 (m, Aliph-CH), 1660 (s, CO), 1610 (s, C=N), 1590 (m, C=C) cm^-1^. ^1^H NMR (400 MHz, DMSO-*d_6_*): δ = 11.40 (s, 1 H, NH), 7.20-7.00 (m, 5 H, Ar-H), 7.06-6.90 (m, 4 H, Ar-H), 6.10 (s, 1 H, Ar-CH), 2.49 (s, 2 H, CH_2_), 2.20 (d, *J* = 16.1 Hz, 1 H, 7-H), 2.05 (d, *J* = 16.1 Hz, 1 H, 7\-H), 1.00 (s, 3 H, CH_3_), 0.98 (s, 3 H, CH_3_). ^13^C NMR (400 MHz, DMSO-*d_6_*): δ = 190.6 (C-6), 141.2 (C-10a), 140.8 (C-9a), 134.6, 134.2 (Ar-C), 130.0 (Ar-C-1\), 130.0, 129.6, 128.4, 128.0 (bz-CH), 127.6 (2 Ph-CH-3\, -5\), 126.8, (2 Ph-CH-2\, -6\) 126.0 (Ph-CH-4\), 110.6 (C-6a), 99.0 (CH-5), 48.0 (C-7), 40.4 (C-9), 32.2 (C-8), 28.4 (CH_3_), 26.8 (CH_3_). EI-MS: *m*/*z* (%) = 343(100) [M^+^], 328 (20), 312 (14), 236 (18), 266 (34), 250 (18), 77 (48). Anal. Calcd for C_22_H_21_N_3_O (343.43): C, 76.94; H, 6.16; N, 12.24. Found: C, 76.80; H, 6.12; N, 12.22.
